# Lipid raft localization of TLR2 and its co-receptors is independent of membrane lipid composition

**DOI:** 10.7717/peerj.4212

**Published:** 2018-01-05

**Authors:** Christine Hellwing, Axel Schoeniger, Claudia Roessler, Anja Leimert, Julia Schumann

**Affiliations:** 1 Clinic for Anesthesiology and Surgical Intensive Care, University Hospital Halle (Saale), Halle (Saale), Germany; 2 Institute of Biochemistry, Faculty of Veterinary Medicine, University of Leipzig, Leipzig, Germany

**Keywords:** TLR2, TLR1, TLR6, Macrophages, Lipid rafts, Membrane lipid composition, PUFA

## Abstract

**Background:**

Toll like receptors (TLRs) are an important and evolutionary conserved class of pattern recognition receptors associated with innate immunity. The recognition of Gram-positive cell wall constituents strongly depends on TLR2. In order to be functional, TLR2 predominantly forms a heterodimer with TLR1 or TLR6 within specialized membrane microdomains, the lipid rafts. The membrane lipid composition and the physicochemical properties of lipid rafts are subject to modification by exogenous fatty acids. Previous investigations of our group provide evidence that macrophage enrichment with polyunsaturated fatty acids (PUFA) induces a reordering of lipid rafts and non-rafts based on the incorporation of supplemented PUFA as well as their elongation and desaturation products.

**Methods:**

In the present study we investigated potential constraining effects of membrane microdomain reorganization on the clustering of TLR2 with its co-receptors TLR1 and TLR6 within lipid rafts. To this end, RAW264.7 macrophages were supplemented with either docosahexaenoic acid (DHA) or arachidonic acid (AA) and analyzed for receptor expression and microdomain localization in context of TLR stimulation.

**Results and Conclusions:**

Our analyses showed that receptor levels and microdomain localization were unchanged by PUFA supplementation. The TLR2 pathway, in contrast to the TLR4 signaling cascade, is not affected by exogenous PUFA at the membrane level.

## Introduction

The innate immune system represents the first barrier against invading pathogenic agents. It is based on the recognition of pathogen-associated molecular patterns (PAMPs) by genetically invariant receptors. This allows the organism to react to microbial invasions immediately. Toll like receptors (TLRs) are an important and evolutionary conserved class of pattern recognition receptors associated of innate immunity. TLRs are preferentially expressed on antigen presenting cells (APCs) such as monocytes/macrophages, dendritic cells, and B cells but are also found on endothelial and epithelial cells (Hallman, Ramet & Ezekowitz, 2001; Wetzler, 2003). The type I transmembrane receptors belong to the IL-1/TLR receptor super family (Buwitt-Beckmann et al., 2006; Palsson-McDermott & O’Neill, 2007). The extracellular domain containing multiple leucine-rich repeats (LRR) is assumed to contribute to ligand binding (Hallman, Ramet & Ezekowitz, 2001; Palsson-McDermott & O’Neill, 2007). The intracellular domain, referred to as the TIR domain due to its homology to the IL-1 receptor, is involved in downstream signaling (Hallman, Ramet & Ezekowitz, 2001; Palsson-McDermott & O’Neill, 2007). Upon ligand binding, the recruitment of TIR-containing cytosolic adapters ultimately leads to the activation of both nuclear factor κB (NFκB) and mitogen-activated protein kinases (MAPKs) (Hallman, Ramet & Ezekowitz, 2001; Frazao, Errante & Condino-Neto, 2013). TLR signaling induces an increase in pro-inflammatory cytokine production and promotes co-stimulatory molecule expression by APCs, which triggers innate immune responses and stimulates acquired immunity.

The best characterized TLR is TLR4, which recognizes PAMPs from Gram-negative bacteria, mainly lipopolysaccharides (LPS). The recognition of Gram-positive cell wall constituents such as bacterial lipopeptides and lipoteichoic acid (LTA) strongly depends on TLR2. In order to be functional, TLR2 predominantly forms heterodimers with TLR1 or TLR6 (Hallman, Ramet & Ezekowitz, 2001; Wetzler, 2003; Buwitt-Beckmann et al., 2006; Palsson-McDermott & O’Neill, 2007; Frazao, Errante & Condino-Neto, 2013). The formation of heterodimers between TLR2 and other TLRs for ligand recognition broadens the range of bacterial structures that serve as TLR2 ligands. At this, TLR2 in combination with TLR1 primarily recognizes triacylated lipopetides, whereas TLR2 in combination with TLR6 recognizes mainly diacylated lipopeptides and LTA (Buwitt-Beckmann et al., 2006; Palsson-McDermott & O’Neill, 2007; Frazao, Errante & Condino-Neto, 2013).

Ligand-triggered interaction of a TLR receptor with its corresponding co-receptor is a prerequisite for initiation of the TLR mediated signaling cascade since it creates a scaffold for recruitment of specific adapter molecules. The presence of LPS or Gram-negative bacteria, for example, induces the interplay between TLR4 and its co-receptor CD14. Of note, this stimulation-dependent association of TLR4 with CD14 has been shown by us and others to take place in lipid rafts (Olsson & Sundler, 2006; Triantafilou et al., 2006; Parker et al., 2008; Wong et al., 2009; Fessler & Parks, 2011; Schoeniger, Fuhrmann & Schumann, 2016). The recruitment of TLR4 and CD14 into lipid rafts upon ligand binding is believed to facilitate the assembly of the signaling complex, as it increases the probability of interaction between these proteins (Nicolau Jr et al., 2006; Fessler & Parks, 2011). Similar to TLR4, TLR2 is described to form heterodimeric clusters with TLR1 and TLR6 that reside in raft microdomains (Triantafilou et al., 2006). In the absence of a ligand it seems that TLR1, TLR2, and TLR6 form weakly bound multimers, which rearrange upon ligand binding to initiate signaling within lipid rafts (Jin et al., 2007).

Lipid raft composition and organization are regulated by several factors, such as the presence of cholesterol and calcium ion balance. Cholesterol plays a fundamental role in the biophysical properties of membranes. It is reported to modulate membrane integrity and fluidity, to participate in bilayer packing, and to contribute to the formation of lipid rafts (Sciacca et al., 2016). Disruption of intracellular calcium homeostasis is described to affect membrane domain organization, vesicular trafficking, and adhesion/fusion processes (Sciacca et al., 2013; Milardi et al., 2014). In doing so both, cholesterol and calcium ions, regulate basic physiological processes und functions of various cells including leukocytes. In fact, immune modulating properties have been described for cholesterol (Fessler, 2016) and calcium ions (Robert et al., 2013).

Another factor known to influence membrane lipid composition and physicochemical properties of lipid rafts is the availability of exogenous fatty acids. Previous investigations of our group provide evidence that cellular enrichment with polyunsaturated fatty acids (PUFA) induces a reordering of membrane domains (Schumann et al., 2011; Hellwing et al., 2018). The profound alterations in the lipid profile of the membrane were observed by both gas chromatographic and mass spectrometric analyses of detergent-free isolated microdomains. These changes are based on the incorporation of supplemented PUFA along with their elongation and desaturation products into lipid rafts and non-rafts (Schumann et al., 2011; Hellwing et al., 2018). The shift towards a higher proportion of highly unsaturated fatty acids induced a displacement of sterically incompatible lipids and proteins from the respective membrane compartments (Hellwing et al., 2018). Thus, there is an extensive reorganization of membrane architecture, which is likely to impact on receptor function and downstream events leading to functional consequences.

Indeed, PUFA supplementation of cells has been shown by us and others to impair the LPS-mediated activation of the transcription factor NFκB (Zhao et al., 2005; Ren & Chung, 2007; Weldon et al., 2007; Chang et al., 2010; Mullen, Loscher & Roche, 2010; Schumann & Fuhrmann, 2010). This corresponds to a suppression of the LPS-mediated differentiation of macrophages into the inflammation-driving M1 phenotype. Both the stimulation-induced respiratory burst and the stimulation-induced production of pro-inflammatory cytokines is diminished due to macrophage enrichment with PUFA (Schoeniger et al., 2011; Adolph et al., 2012). Consequently, infusion of PUFA in parenteral nutrition represents an established clinical routine, which is reported to result in genuine benefits in the treatment of humans suffering from immunological imbalances such as sepsis patients (Wanten & Calder, 2007).

Despite the clinical usage of PUFA the underlying mechanisms have not been adequately clarified yet. PUFA might influence immune defense by various ways. Firstly, some PUFA serve as precursors of the immune modulating eicosanoids and resolvins (Calder, 2008; Serhan, Chiang & Van Dyke, 2008). Secondly, PUFA are known ligands of certain nuclear receptors including the peroxisome proliferator-activated receptors (PPARs) or the G protein-coupled receptor 120 (GPR120) (Calder, 2008; Oh et al., 2010). Thirdly, as stated above, PUFA play a pivotal role in membrane microdomain organization by altering plasma membrane topography and the spatial organization of cholesterol and phosphatidylinositol-bisphosphates (Kucerka et al., 2010; Hou et al., 2016). In doing so PUFA are able to affect cellular signal transduction. In fact, we recently observed a marked decrease in stimulation-induced clustering of TLR4 and CD14 in lipid rafts due to PUFA incorporation into macrophage membrane domains (Schoeniger, Fuhrmann & Schumann, 2016). This provides evidence that the membrane domain localization of TLR4 and its co-receptor CD14 not only depends on activation status but also on membrane lipid composition.

For the TLR2 signaling cascade, however, the situation is less clear. Bearing in mind the frequency of PUFA treatment in clinical praxis and with regard to the significance of infections caused by gram-positive pathogens, e.g., *Staphylococcus aureus*, this is an unsatisfactory situation. In the present study we therefore investigated potential constraining effects of membrane microdomain reorganization on the clustering of TLR2 with its co-receptors TLR1 and TLR6 within lipid rafts.

## Materials and Methods

### Materials

Chemicals/reagents were acquired from Sigma-Aldrich (Taufkirchen, Germany) except where otherwise specified. HEPES (25 mmol/L)-buffered RPMI 1640 culture medium including 300 mg/L L glutamine was obtained from PAN-Biotech GmbH (Aidenbach, Germany) and cell culture flasks were purchased from Greiner Bio-One (Frickenhausen, Germany).

### Culturing, fatty acid supplementation, and stimulation of cells

The murine cell line RAW264.7 (ATCC^®^ number TIB-71) was used as a model of monocytes/macrophages. Culturing of RAW264.7 was performed at 37 °C and 5% CO_2_ in a humidified atmosphere using RPMI 1640 medium containing 4.5 g/L glucose, 5% v/v FCS, and 0.1% ethanol. For fatty acid supplementation of cells either docosahexaenoic acid (DHA, C22:6n3) or arachidonic acid (AA, C20:4n6) (Biotrend, Köln, Germany) was included in the culture medium in concentrations of 15 µmol/L using ethanol as a vehicle (0.1% v/v final ethanol concentration) for a period of 72 h. For stimulation of cells either lipoteichoic acid (LTA, 0.5 µg/mL, from *Staphylococcus aureus*) or viable *Rhodococcus equi* (ATCC^®^ number 6939, non-virulent or ATCC^®^ number 33701, virulent; bacterium/cell ratio 0.1:1) were included in the culture medium in either the last 15 min or the last 24 h of fatty acid supplementation.

### Digital droplet PCR

RAW264.7 were cultured, supplemented, and stimulated as described above. Gene expression was analyzed using the QX200 Droplet Digital PCR (ddPCR) system from Bio-Rad (Munich, Germany) according to the manufacturer’s instructions. Total RNA was extracted using the InviTrap Spin Universal RNA Mini Kit (Stratec Biomedical, Birkenfeld, Germany). Complementary DNA was synthesized using the qScript cDNA SuperMix (Quanta BioSciences, Gaithersburg, MD, USA). Gene expression of TLR1, TLR2, and TLR6 was analyzed by quantitative real-time PCR using the ddPCR EvaGreen Supermix (Bio-Rad, Munich, Germany). Primer sequences and thermal cycling conditions used can be found in Table 1. Negative controls, i.e., no template control, and no reverse transcriptase control, were performed for each run. ddPCR data were analyzed using QuantaSoft analysis software (Bio-Rad, Munich, Germany), and the quantification of the target molecule was presented as the number of copies per µg total RNA used. Measurements were performed in triplicates and are representatives of three independent experiments for each combination of fatty acid and stimulant.

**Table 1:** Primer sequences and thermal cycling conditions. Target, primer sequence, product size, annealing temperature (X) and elongation time (Y) used for quantitative ddPCR. Cycling conditions were as follows: initial denaturation for 3 min at 95 °C, followed by 44 cycles of 10 s denaturation at 95 °C, 10 s annealing at X °C, and extension at 72 °C for Y s.

Target	Primer sequence (5′→3′)	Product size (bp)	Annealing temperature (°C)	Extension time (s)
TLR1	GTTGGTGAAGAACTCAGGCG GTAGGTCCTTGGGCACTCTG	258	54	20
TLR2	GAGGTGCGGACTGTTTCCTT GAGCCAAAGAGCTCGTAGCA	174	55	15
TLR6	ATGGCACAGCGGACTTACTT AGAGCCCAGGTTGACAGTTTATT	217	59	20

### Flow cytometry

RAW264.7 were cultured, supplemented, and stimulated as described above. Cells were stained for the cell receptors TLR1, TLR2, and TLR6 and fixed in 1% paraformaldehyde in PBS. At this, staining was performed with anti-TLR1-PE (Thermo Fisher Scientific, Dreieich, Germany), anti-TLR2-FITC (BioLegend, Fell, Germany) plus anti-TLR6-APC (R & D Systems, Wiesbaden, Germany). The Fc receptors were blocked in all experiments by incubating the cells with FcR blocking reagent (Miltenyi Biotech, Bergisch Gladbach, Germany). The specificity of all antibodies used was verified by appropriate isotop controls. Cells were analyzed on a FacsCalibur using Cellquest Pro software (all Becton Dickinson, Heidelberg, Germany). Analyses were performed in duplicates and are representatives of four independent experiments for each combination of fatty acid and stimulant of cells.

### Fluorescence microscopy

RAW264.7 were cultured on sterile coverslips (Menzel GmbH, Braunschweig, Germany) in 12 well plates (TPP Techno Plastic Products AG, Trasadingen, Switzerland), supplemented, and stimulated as described above. Cells were fixed for 10 min in 4% paraformaldehyde and rinsed in PBS twice. Non-specific binding was blocked for 1 h at 37 °C with 1% BSA in PBS. Cells were incubated with species-specific primary antibodies against GM1 (Bioss, Woburn, Massachusetts, USA), TLR1 (R & D Systems, Wiesbaden, Germany), TLR2 (BioLegend, Fell, Germany), and TLR6 (R & D Systems, Wiesbaden, Germany) in a humidified chamber for 1 h at ambient temperature. Subsequently, cells were rinsed in PBS thrice and incubated in the dark with appropriate secondary antibodies conjugated with Alexa Fluor 350, Alexa Fluor 488, and Alexa Fluor 594, respectively (Thermo Fisher Scientific, Dreieich, Germany) for 1 h in a humidified chamber at ambient temperature. The coverslips with the labeled cells were rinsed in PBS thrice, mounted onto glass slides using DAKO fluorescence mounting medium (Dako Deutschland GmbH, Hamburg, Germany), and examined with a BZ-9000 fluorescence microscope (Keyence, Neu-Isenburg, Germany). Microscopic images were taken from each quadrant of a coverslip and analyzed using the public domain software ImageJ (Version 1.50i). Co-localization was quantified using Manders’ (M1) coefficient as previously described (Manders, Verbeek & Aten, 1993). Analyses were performed in quadruplicate and are representative of four independent experiments for each combination of fatty acid and stimulant.

### Statistical analysis

Data are shown as mean ± standard deviation (S.D.). In order to identify significant differences between means a one-way analysis of variance followed by Holm-Sidak corrected multiple comparison was performed. The statistical analysis was carried out with GraphPad Prism 6 (GaphPad Software, La Jolla, CA, USA). In all cases, *p* < 0.05 was considered to indicate significant differences.

## Results

### TLR1, TLR2, and TLR6 expression is hardly influenced by PUFA supplementation

The impact of macrophage supplementation with unsaturated fatty acids in the absence or presence of a TLR2 ligand on TLR1, TLR2, and TLR6 receptor expression was assessed by ddPCR on RNA level and by flow cytometry on protein level.

**Figure 1:**
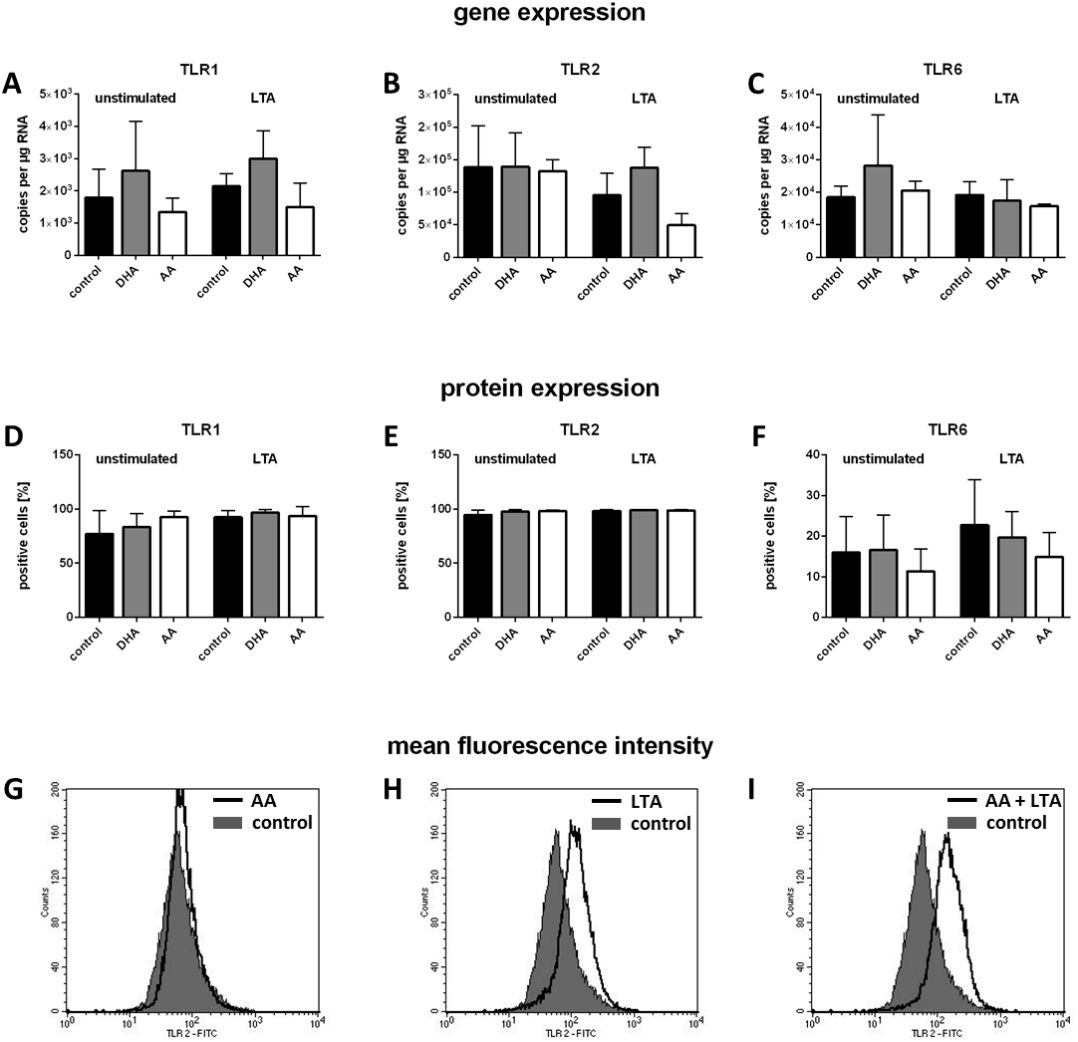
Gene and protein expression of TLR1, TLR2, and TLR6. RAW264.7 were cultured in basic medium (RPMI 1640 containing 4.5 g/L glucose, 5% v/v FCS, 0.1% v/v ethanol) supplemented with either docosahexaenoic acid (DHA) or arachidonic acid (AA) in a concentration of 15 µM for 72 h. Stimulation was performed by addition of LTA (0.5 µg/mL) to the culture medium in the last 24 h of incubation. Gene expression was analyzed by ddPCR (absolute quantification) (*N* = 3, *n* = 3); protein expression was analyzed by flow cytometry (*N* = 4, *n* = 2). Data are expressed as mean ± S.D. (A–C) TLR1/TLR2/TLR6 mRNA copies per µg isolated total RNA. (D–F) Percentage of TLR1/TLR2/TLR6 positive cells. (G–I) Representative images of mean fluorescence intensity.

PUFA enrichment of the cell culture medium had no effect on receptor mRNA expression. Both DHA and AA did not affect the transcription rate of the TLR1, TLR2 and TLR6 genes in unstimulated as well as stimulated RAW264.7. There were no significant differences in receptor gene copies per µg of isolated total RNA between RAW264.7 control cells and cells treated with DHA or AA (Figs. 1A–1C). Stimulation with LTA did not change the result (Figs. 1A–1C).

Flow cytometric analysis showed that almost all RAW264.7 cells expressed the receptors TLR1 and TLR2 on their surface, whereas the percentage of TLR6-positive cells was substantially lower (Figs. 1D–1F). These proportions did not change following LTA stimulation and/or PUFA supplementation (Figs. 1D–1F). The number of receptors expressed by a single cell (as determined by mean fluorescence intensity) slightly increased for TLR1 and TLR6 following LTA stimulation (supplementary data). In the case of TLR2 there was a significant increase, and this stimulation-dependent effect was enhanced by PUFA treatment of the macrophages (Figs. 1G–1I; AA effect is shown exemplary).

### Lipid raft localization of TLR1, TLR2, and TLR6 is independent of the macrophage membrane lipid composition

The membrane microdomain distribution of TLR1, TLR2, and TLR6 was assessed by fluorescence microscopic analysis of receptor co-localization with the lipid raft marker ganglioside GM1. Microscopic data were quantified by means of the Manders’ (M1) coefficient. The M1 coefficient is proportional to the amount of fluorescence of the co-localizing pixels in each color channel (Manders, Verbeek & Aten, 1993). Values range from 0 (no co-localization) to 1 (perfect co-localization). Since the M1 coefficient is not sensitive to differences in pixel intensities of an image, it is not affected by potential cellular variations in receptor expression (Manders, Verbeek & Aten, 1993).

**Figure 2:**
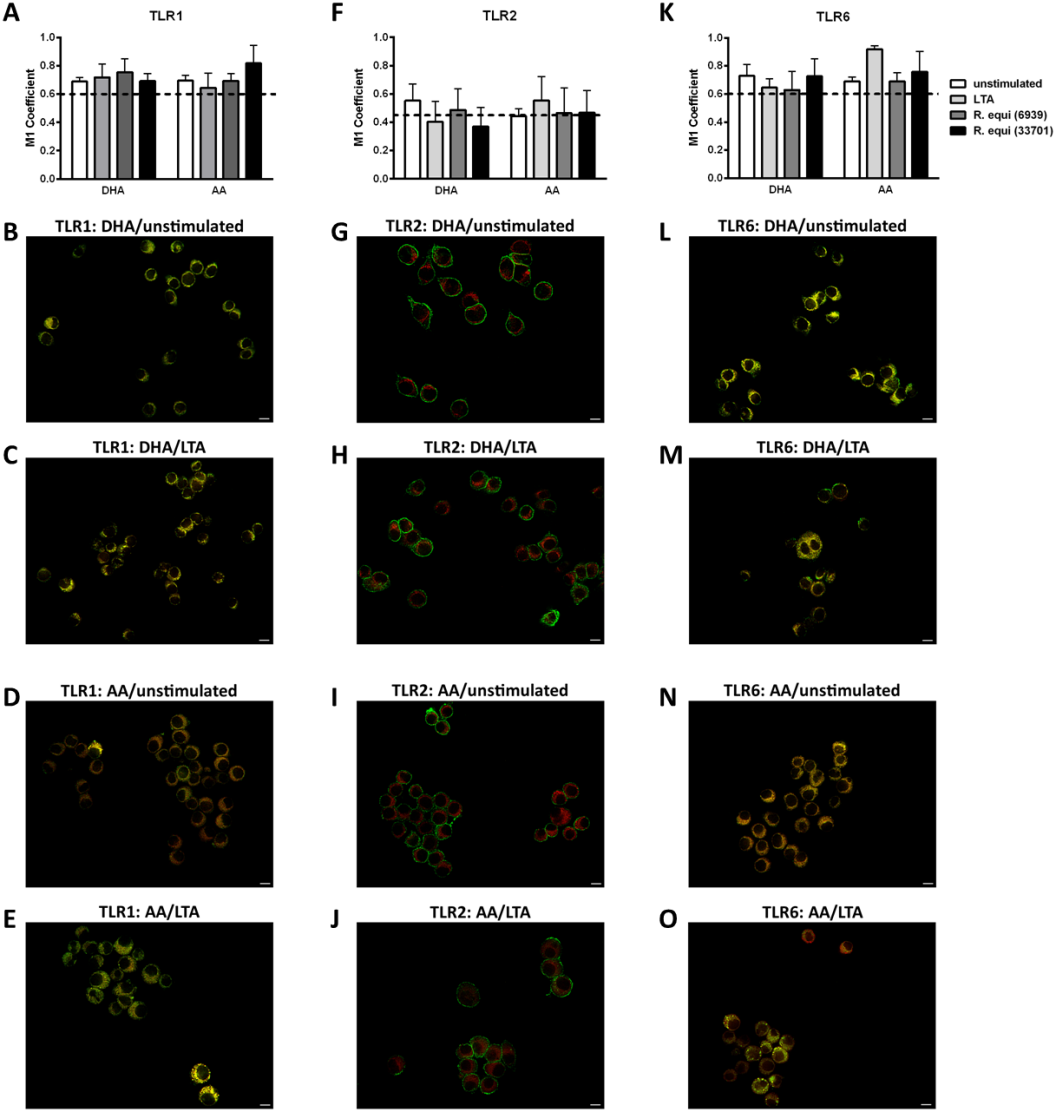
Co-localization of TLR1, TLR2 or TLR6 with the raft marker GM1. RAW264.7 were cultured in basic medium (RPMI 1640 containing 4.5 g/L glucose, 5% v/v FCS, 0.1% v/v ethanol) supplemented with either docosahexaenoic acid (DHA) or arachidonic acid (AA) in a concentration of 15 µM for 72 h. Stimulation was performed by addition of LTA (0.5 µg/mL) or viable *Rhodococcus equi* (MOI 0.1; ATCC^®^ number 6939 = non-virulent, ATCC^®^ number 33701 = virulent) to the culture medium in the last 24 h of incubation. Co-localization of TLR1, TLR2, and TLR6 with GM1 was analyzed by indirect immunofluorescence microscopy. Quantification was performed by calculating the Manders’ (M1) coefficient using the JACoP plugin of ImageJ. Data are expressed as mean ± S.D. (*N* = 4, *n* = 4). The dotted line represents the respective M1 coefficient of unsupplemented and unstimulated RAW264.7. In the representative images TLR1, TLR2, and TLR6 respectively are labeled in green; GM1 is labeled in red. Scale bar represents 10 µm. (A–E) M1 coefficients and representative images of TLR1-GM1 co-localization. (F–J) M1 coefficients and representative images of TLR2-GM1 co-localization. (K–O) M1 coefficients and representative images of TLR6-GM1 co-localization.

For RAW264.7 cultured in basic medium M1 coefficients of 0.62 for TLR1, 0.45 for TLR2, and 0.61 for TLR6 were found (Fig. 2, dotted line). These values were slightly increased by PUFA enrichment of the macrophage membrane microdomains. DHA-supplemented RAW264.7 displayed M1 coefficients of 0.69 for TLR1 as well as TLR6, and 0.55 for TLR2 (Fig. 2). Likewise, for AA-supplemented RAW264.7 M1 coefficients of 0.70 for TLR1, 0.44 for TLR2, and 0.73 for TLR6 were observed (Fig. 2). Incorporation of unsaturated fatty acids into plasma membrane lipid rafts and non-rafts in absence of a TLR2 ligand therefore seems not to affect the microdomain localization of the receptors TLR1, TLR2, and TLR6.

Next, we examined the combined effects of PUFA supplementation and stimulation. Beside the well described TLR2 ligand LTA, complex biological stimuli, namely viable gram-positive *R. equi* microorganisms, were used. M1 coefficients for DHA-enriched RAW264.7 were in the range of 0.69 to 0.75 for TLR1, 0.37 to 0.49 for TLR2, and 0.63 to 0.73 for TLR6 following stimulation (Fig. 2). For RAW264.7 previously supplemented with AA stimulation-dependent M1 coefficients in the range of 0.64 to 0.81 for TLR1, 0.47 to 0.55 for TLR2, and 0.69 to 0.92 for TLR6 were detected (Fig. 2). The microscopic data illustrate that the receptors remained in (TLR1 and TLR6) or close to (TLR2) lipid rafts (Fig. 2).

In addition to the 24 h stimulation period (long-term treatment) discussed above the fluorescence microscopy was also performed with short-term treated RAW264.7 (15 min stimulation period) in order to investigate potential receptor redistribution immediately following macrophage-stimulant interaction (Supplementary Information). With this experimental setup and in accordance with the aforementioned data no significant changes in M1 coefficients were observed thus underlining the high persistence of TLR1, TLR2, and TLR6 localization in membrane microdomains.

## Discussion

Activation of the TLR2 pathway induces the polarization of a macrophage into the inflammation-driving M1 phenotype. M1 macrophages contribute to immune defense by the synthesis and release of pro-inflammatory cytokines as well as the production of reactive oxygen and nitrogen intermediates (ROI/RNI). Supplementation of macrophages with unsaturated fatty acids prior to TLR2 stimulation, however, interferes with M1 polarization. Previous investigations of our group demonstrate that the activation-induced production of pro-inflammatory cytokines, ROI, and RNI is reduced in PUFA-enriched macrophages compared to macrophages cultured under basic conditions (Schoeniger et al., 2011; Adolph et al., 2012). In addition, our gas chromatographic and mass spectrometric analyses of detergent-free isolated membrane microdomains provide evidence that supplementation of macrophages with exogenous PUFA goes along with an enrichment of the membrane with unsaturated fatty acids (Schumann et al., 2011; Hellwing et al., 2018). We therefore speculated that the reorganization of lipid rafts and non-rafts in PUFA treated macrophages and the resulting changes in membrane domain physicochemical properties might have an impact on the interaction of TLR2 receptor with its co-receptors TLR1 and TLR6, respectively. To this end, RAW264.7 macrophages were supplemented with either DHA or AA in a physiologically relevant concentration of 15 µM and analyzed for receptor expression and microdomain localization in context of TLR stimulation. Periods of supplementation and stimulation were chosen in accordance with our previous investigations and were proven there to result in a membrane fatty acid steady state as well as reproducible effects on macrophage functionality. Biological relevance of the results was further improved by using viable gram-positive microorganisms as TLR ligands.

Our investigations indicate that PUFA enrichment of macrophages does not alter the mRNA expression of TLR1, TLR2, and TLR6. Likewise, no impact of PUFA on the percentage of cells positive for TLR1, TLR2, and TLR6 was found by flow cytometric analysis. A significant PUFA effect was only seen in stimulated RAW264.7 on the single cell level. Supplementation with DHA or AA further increased the stimulation-dependent induction of TLR2 receptor expression by a single cell. Based on these data it seems unlikely that the impairment of TLR-mediated activation of PUFA-supplemented macrophages is due to a modulation of TLR expression. To our knowledge this is the first study investigating the effects of exogenous fatty acids on TLR1, TLR2, and TLR6 expression levels. Nevertheless, unsaturated fatty acids are described to suppress TLR4 gene expression in enterocytes and intestinal cells but not in adipose stem cells and HEK cells (Lu et al., 2007; Hsueh et al., 2011; Liu et al., 2012; Murumalla et al., 2012). In a previous study of our group, all PUFA tested, except DHA, failed to affect TLR4 mRNA levels of RAW264.7 macrophages (Schoeniger, Fuhrmann & Schumann, 2016). Thus, it seems common to TLR receptors that there synthesis is hardly influenced by exogenous fatty acids.

Regarding membrane microdomain distribution we found no effect of macrophage PUFA supplementation for TLR1, TLR2, and TLR6. Irrespective of the absence or presence of a TLR2 ligand, PUFA were not able to modulate the microdomain localization of the receptors. Co-localization of TLR1, TLR2, and TLR6 with the lipid raft ganglioside GM1 remained unchanged for different membrane lipid compositions. Our data indicate that the TLR2 pathway is not influenced by PUFA supplementation at the membrane level.

Furthermore, according to our data TLR1, TLR2, and TLR6 are highly associated to lipid rafts. This is in line with previous observations in the literature. TLR2 and its co-receptors TLR1 and TLR6 have been shown to form multimers even in the absence of a receptor ligand (Jin et al., 2007). Ligand binding is proposed to induce a rearrangement of these pre-existing multimers bringing the C termini into closer proximity (Jin et al., 2007). It appears plausible that these remodeling processes are less sensitive to microdomain reorganization by exogenous fatty acids.

In prior research using an identical experimental design we provided evidence that PUFA markedly alter the lipid raft localization of TLR4 and its co-receptor CD14. In unstimulated RAW264.7, PUFA supplementation resulted in a significantly increased co-localization of TLR4 and CD14 with the lipid raft marker GM1 (Schoeniger, Fuhrmann & Schumann, 2016). In the presence of a TLR4 ligand, however, PUFA were found to inhibit the stimulation-induced recruitment of TLR4 and CD14 into lipid rafts (Schoeniger, Fuhrmann & Schumann, 2016). Hence, the observations made for TLR4 contrast with our present data obtained for the TLR2 signaling cascade. One possible explanation for this finding is that TLR4 and its co-receptor CD14 have been shown by us and others to reside outside of lipid rafts in the absence of an appropriate ligand, and to be recruited into the microdomains upon stimulation (Olsson & Sundler, 2006; Triantafilou et al., 2006; Parker et al., 2008; Wong et al., 2009; Fessler & Parks, 2011; Schoeniger, Fuhrmann & Schumann, 2016). This process is very likely to depend on the physicochemical properties of membranes and, hence, to be altered by PUFA enrichment of lipid rafts.

Clearly, further research is needed to identify the molecular mechanisms underlying the actions of PUFA. This is particularly true since the fatty acids investigated in our study are used in the medical care of severely ill people. Emphasis should be given to the direct influence of DHA and AA on immunologically relevant receptors such as PPARs or GPR120. Another important issue is the role of fatty acid derivatives in TLR-mediated signaling. Both PUFA tested in our study can be converted into potent immune modulators such as pro-inflammatory prostaglandins and anti-inflammatory resolvins (Calder, 2008; Serhan, Chiang & Van Dyke, 2008). It is well known that prostaglandins and resolvins differ in their immunological activity depending on the PUFA they derive from (Calder, 2008; Serhan, Chiang & Van Dyke, 2008). However, the regulation of TLR expression by these fatty acid derivatives and the interdependence of TLR- and prostaglandin/resolvin-mediated signaling cascades remains an area that has been largely neglected in past research.

## Conclusions

Our data confirm previous findings that TLR2/TLR1- and TLR2/TLR6-heterodimerization takes place in lipid rafts (Triantafilou et al., 2006). This membrane domain localization remains unaffected by PUFA enrichment. Thus, TLR2 and its dimerization partners TLR1 and TLR6, unlike TLR4 and CD14, reside in lipid rafts irrespective of membrane lipid composition.

##  Supplemental Information

10.7717/peerj.4212/supp-1 Data S1 Raw data Overview in table form of the raw data displayed in Figs. 1 and 2.

10.7717/peerj.4212/supp-2 Supplemental Information 1 Mean fluorescence intensity TLR1 and TLR6 RAW264.7 were cultured in basic medium (RPMI 1640 containing 4.5 g/L glucose, 5% v/v FCS, 0.1% v/v ethanol) supplemented with arachidonic acid (AA) in a concentration of 15 µM for 72 h. Stimulation was performed by addition of LTA (0.5 µg/mL) to the culture medium in the last 24 h of incubation. Protein expression was analyzed by flow cytometry: Representative images of mean fluorescence intensity of TLR1 and TLR6 respectively.

10.7717/peerj.4212/supp-3 Supplemental Information 2 TLR1 AA LTA RAW264.7 were cultured in basic medium (RPMI 1640 containing 4.5 g/L glucose, 5% v/v FCS, 0.1% v/v ethanol) supplemented with arachidonic acid (AA) in a concentration of 15 µM for 72 h. Stimulation was performed by addition of LTA (0.5 µg/mL) to the culture medium in the last 24 h of incubation. Co-localization of TLR1 with GM1 was analyzed by indirect immunofluorescence microscopy. In the representative image TLR1 is labeled in green; GM1 is labeled in red. Scale bar represents 10 µm.

10.7717/peerj.4212/supp-4 Supplemental Information 3 TLR1 AA unstimulated RAW264.7 were cultured in basic medium (RPMI 1640 containing 4.5 g/L glucose, 5% v/v FCS, 0.1% v/v ethanol) supplemented with arachidonic acid (AA) in a concentration of 15 µM for 72 h. Co-localization of TLR1 with GM1 was analyzed by indirect immunofluorescence microscopy. In the representative image TLR1 is labeled in green; GM1 is labeled in red. Scale bar represents 10 µm.

10.7717/peerj.4212/supp-5 Supplemental Information 4 TLR1 DHA LTA RAW264.7 were cultured in basic medium (RPMI 1640 containing 4.5 g/L glucose, 5% v/v FCS, 0.1% v/v ethanol) supplemented with docosahexaenoic acid (DHA) in a concentration of 15 µM for 72 h. Stimulation was performed by addition of LTA (0.5 µg/mL) to the culture medium in the last 24 h of incubation. Co-localization of TLR1 with GM1 was analyzed by indirect immunofluorescence microscopy. In the representative image TLR1 is labeled in green; GM1 is labeled in red. Scale bar represents 10 µm.

10.7717/peerj.4212/supp-6 Supplemental Information 5 TLR1 DHA unstimulated RAW264.7 were cultured in basic medium (RPMI 1640 containing 4.5 g/L glucose, 5% v/v FCS, 0.1% v/v ethanol) supplemented with docosahexaenoic acid (DHA) in a concentration of 15 µM for 72 h. Co-localization of TLR1 with GM1 was analyzed by indirect immunofluorescence microscopy. In the representative images TLR1 is labeled in green; GM1 is labeled in red. Scale bar represents 10 µm.

10.7717/peerj.4212/supp-7 Supplemental Information 6 TLR2 AA LTA RAW264.7 were cultured in basic medium (RPMI 1640 containing 4.5 g/L glucose, 5% v/v FCS, 0.1% v/v ethanol) supplemented with arachidonic acid (AA) in a concentration of 15 µM for 72 h. Stimulation was performed by addition of LTA (0.5 µg/mL) to the culture medium in the last 24 h of incubation. Co-localization of TLR2 with GM1 was analyzed by indirect immunofluorescence microscopy. In the representative image TLR2 is labeled in green; GM1 is labeled in red. Scale bar represents 10 µm.

10.7717/peerj.4212/supp-8 Supplemental Information 7 TLR2 AA unstimulated RAW264.7 were cultured in basic medium (RPMI 1640 containing 4.5 g/L glucose, 5% v/v FCS, 0.1% v/v ethanol) supplemented with arachidonic acid (AA) in a concentration of 15 µM for 72 h. Co-localization of TLR2 with GM1 was analyzed by indirect immunofluorescence microscopy. In the representative image TLR2 is labeled in green; GM1 is labeled in red. Scale bar represents 10 µm.

10.7717/peerj.4212/supp-9 Supplemental Information 8 TLR2 DHA LTA RAW264.7 were cultured in basic medium (RPMI 1640 containing 4.5 g/L glucose, 5% v/v FCS, 0.1% v/v ethanol) supplemented with docosahexaenoic acid (DHA) in a concentration of 15 µM for 72 h. Stimulation was performed by addition of LTA (0.5 µg/mL) to the culture medium in the last 24 h of incubation. Co-localization of TLR2 with GM1 was analyzed by indirect immunofluorescence microscopy. In the representative image TLR2 is labeled in green; GM1 is labeled in red. Scale bar represents 10 µm.

10.7717/peerj.4212/supp-10 Supplemental Information 9 TLR2 DHA unstimulated RAW264.7 were cultured in basic medium (RPMI 1640 containing 4.5 g/L glucose, 5% v/v FCS, 0.1% v/v ethanol) supplemented with docosahexaenoic acid (DHA) in a concentration of 15 µM for 72 h. Co-localization of TLR2 with GM1 was analyzed by indirect immunofluorescence microscopy. In the representative image TLR2, is labeled in green; GM1 is labeled in red. Scale bar represents 10 µm.

10.7717/peerj.4212/supp-11 Supplemental Information 10 TLR6 AA LTA RAW264.7 were cultured in basic medium (RPMI 1640 containing 4.5 g/L glucose, 5% v/v FCS, 0.1% v/v ethanol) supplemented with arachidonic acid (AA) in a concentration of 15 µM for 72 h. Stimulation was performed by addition of LTA (0.5 µg/mL) to the culture medium in the last 24 h of incubation. Co-localization of TLR6 with GM1 was analyzed by indirect immunofluorescence microscopy. In the representative imags TLR6 is labeled in green; GM1 is labeled in red. Scale bar represents 10 µm.

10.7717/peerj.4212/supp-12 Supplemental Information 11 TLR6 AA unstimulated RAW264.7 were cultured in basic medium (RPMI 1640 containing 4.5 g/L glucose, 5% v/v FCS, 0.1% v/v ethanol) supplemented with arachidonic acid (AA) in a concentration of 15 µM for 72 h. Co-localization of TLR6 with GM1 was analyzed by indirect immunofluorescence microscopy. In the representative images TLR6 is labeled in green; GM1 is labeled in red. Scale bar represents 10 µm.

10.7717/peerj.4212/supp-13 Supplemental Information 12 TLR6 DHA LTA RAW264.7 were cultured in basic medium (RPMI 1640 containing 4.5 g/L glucose, 5% v/v FCS, 0.1% v/v ethanol) supplemented with docosahexaenoic acid (DHA) in a concentration of 15 µM for 72 h. Stimulation was performed by addition of LTA (0.5 µg/mL) to the culture medium in the last 24 h of incubation. Co-localization of TLR6 with GM1 was analyzed by indirect immunofluorescence microscopy. In the representative image TLR6 is labeled in green; GM1 is labeled in red. Scale bar represents 10 µm.

10.7717/peerj.4212/supp-14 Supplemental Information 13 TLR6 DHA unstimulated RAW264.7 were cultured in basic medium (RPMI 1640 containing 4.5 g/L glucose, 5% v/v FCS, 0.1% v/v ethanol) supplemented with docosahexaenoic acid (DHA) in a concentration of 15 µM for 72 h. Co-localization of TLR6 with GM1 was analyzed by indirect immunofluorescence microscopy. In the representative image TLR6 is labeled in green; GM1 is labeled in red. Scale bar represents 10 µm.

10.7717/peerj.4212/supp-15 Supplemental Information 14 TLR localization at 15 min RAW264.7 were cultured in basic medium (RPMI 1640 containing 4.5 g/L glucose, 5% v/v FCS, 0.1% v/v ethanol). Stimulation was performed by addition of LTA (0.5 µg/mL) to the culture medium for 15 min. Co-localization of TLR1, TLR2 or TLR6 with GM1 was analyzed by indirect immunofluorescence microscopy. In the representative images the TLRs are labeled in green; GM1 is labeled in red.

## Additional Information and Declarations

### Data Availability

The following information was supplied regarding data availability: The raw data has been provided as a Supplemental File.
