# Supplementary figures and images for: Lipid raft localization of TLR2 and its co-receptors is independent of membrane lipid composition
Source: PeerJ. 2018 Jan 5;6:e4212. doi: 10.7717/peerj.4212 (PMC5757419; doi:10.7717/peerj.4212)

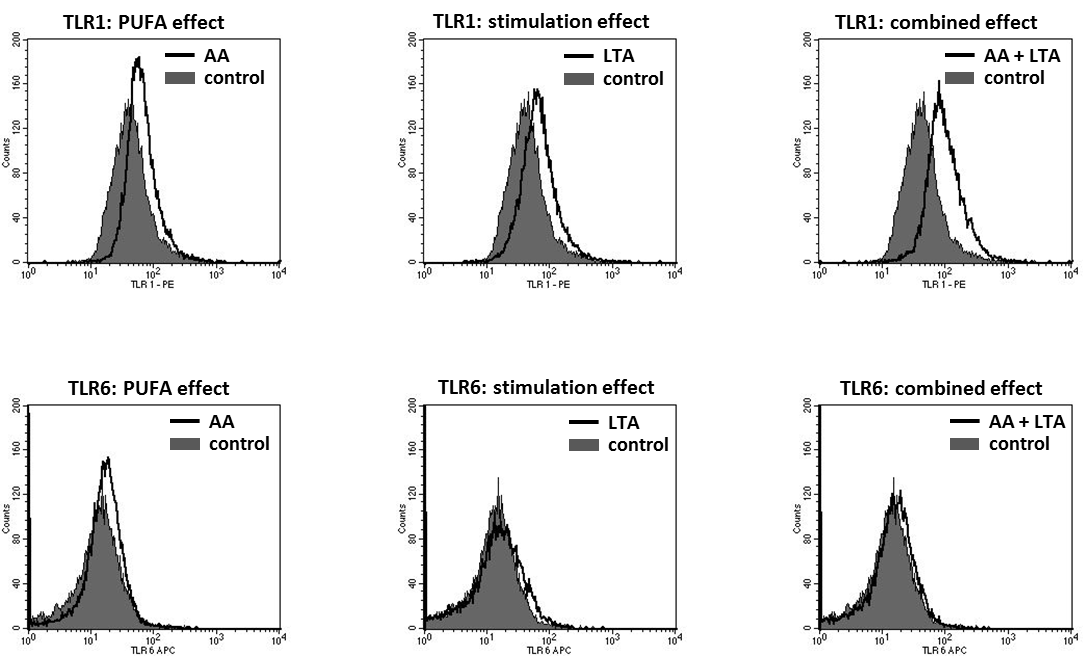

Supplement: Supplemental Information 1 — RAW264.7 were cultured in basic medium (RPMI 1640 containing 4.5 g/L glucose, 5% v/v FCS, 0.1% v/v ethanol) supplemented with arachidonic acid (AA) in a concentration of 15 µM for 72 h. Stimulation was performed by addition of LTA (0.5 µg/mL) to the culture medium in the last 24 h of incubation. Protein expression was analyzed by flow cytometry: Representative images of mean fluorescence intensity of TLR1 and TLR6 respectively. [file peerj-06-4212-s002.png]

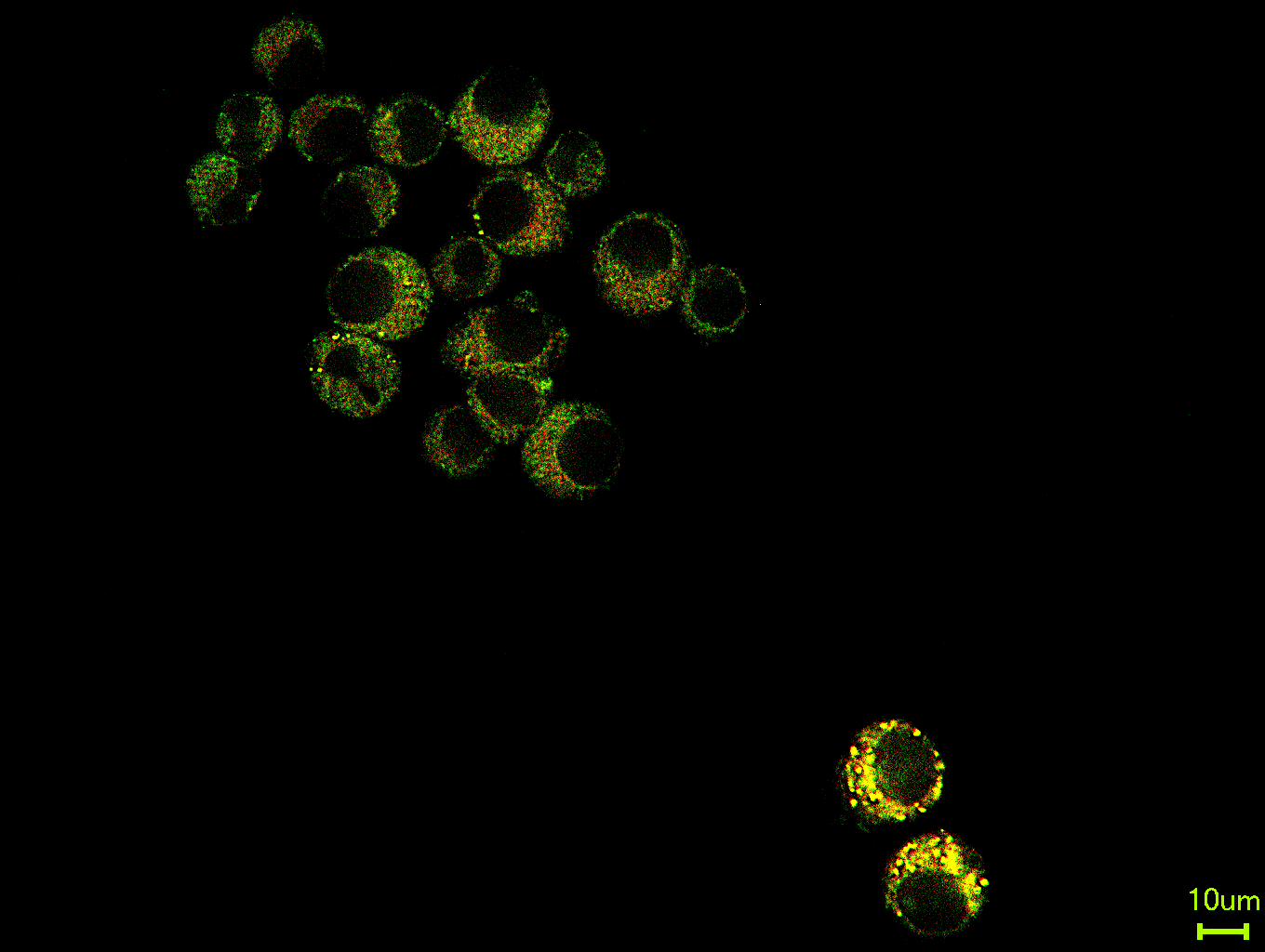

Supplement: Supplemental Information 2 — RAW264.7 were cultured in basic medium (RPMI 1640 containing 4.5 g/L glucose, 5% v/v FCS, 0.1% v/v ethanol) supplemented with arachidonic acid (AA) in a concentration of 15 µM for 72 h. Stimulation was performed by addition of LTA (0.5 µg/mL) to the culture medium in the last 24 h of incubation. Co-localization of TLR1 with GM1 was analyzed by indirect immunofluorescence microscopy. In the representative image TLR1 is labeled in green; GM1 is labeled in red. Scale bar represents 10 µm. [file peerj-06-4212-s003.png]

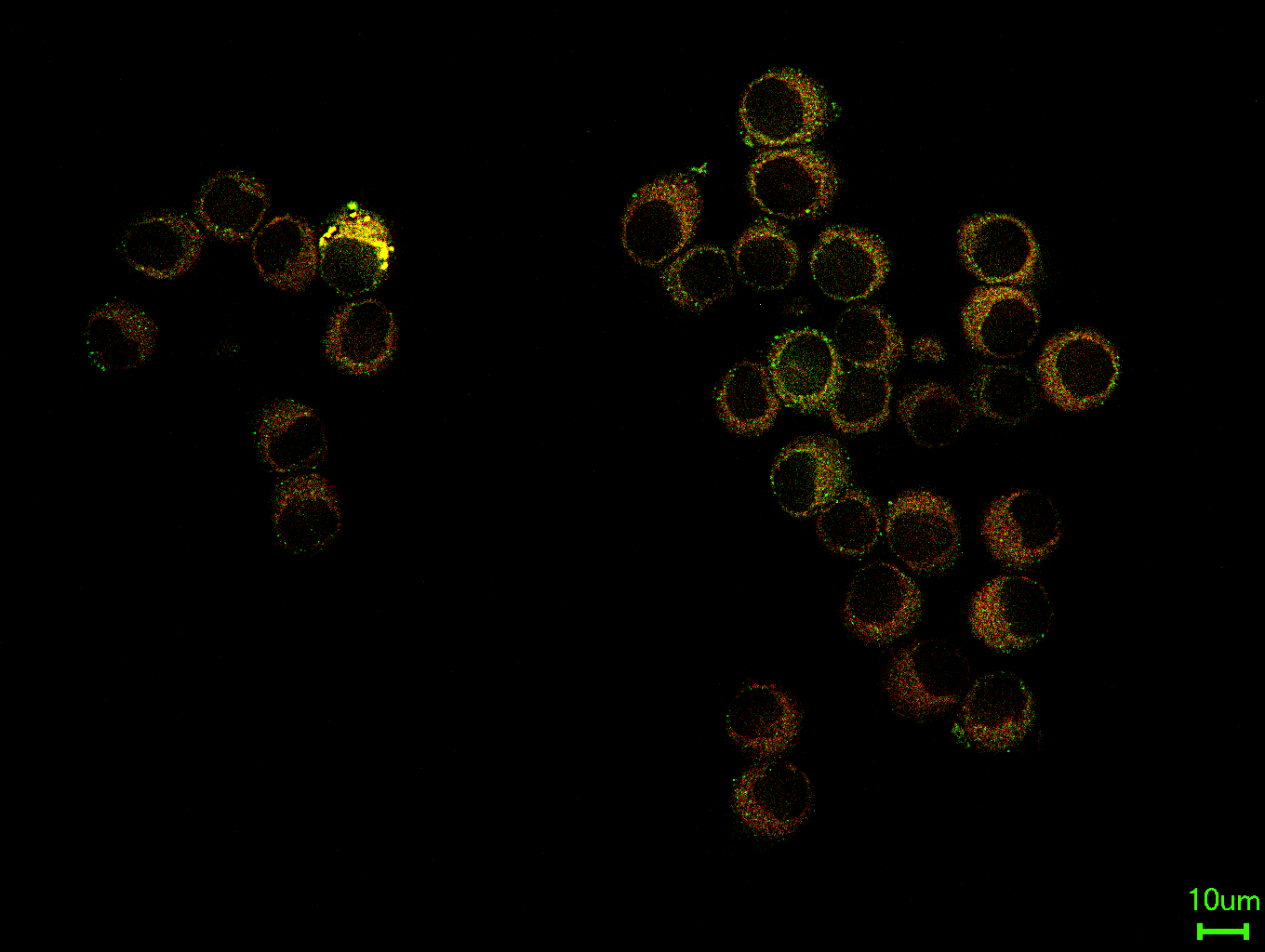

Supplement: Supplemental Information 3 — RAW264.7 were cultured in basic medium (RPMI 1640 containing 4.5 g/L glucose, 5% v/v FCS, 0.1% v/v ethanol) supplemented with arachidonic acid (AA) in a concentration of 15 µM for 72 h. Co-localization of TLR1 with GM1 was analyzed by indirect immunofluorescence microscopy. In the representative image TLR1 is labeled in green; GM1 is labeled in red. Scale bar represents 10 µm. [file peerj-06-4212-s004.png]

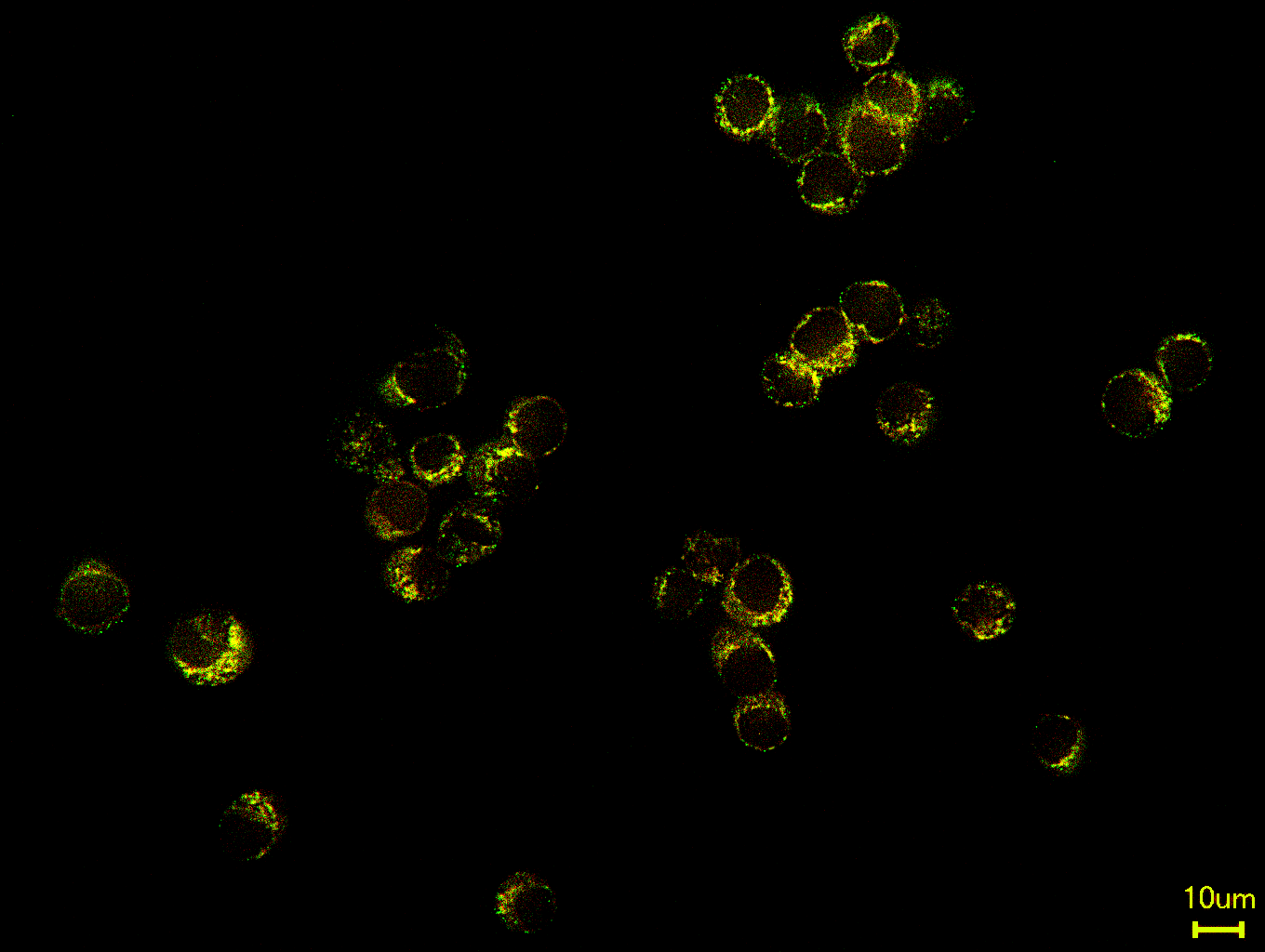

Supplement: Supplemental Information 4 — RAW264.7 were cultured in basic medium (RPMI 1640 containing 4.5 g/L glucose, 5% v/v FCS, 0.1% v/v ethanol) supplemented with docosahexaenoic acid (DHA) in a concentration of 15 µM for 72 h. Stimulation was performed by addition of LTA (0.5 µg/mL) to the culture medium in the last 24 h of incubation. Co-localization of TLR1 with GM1 was analyzed by indirect immunofluorescence microscopy. In the representative image TLR1 is labeled in green; GM1 is labeled in red. Scale bar represents 10 µm. [file peerj-06-4212-s005.png]

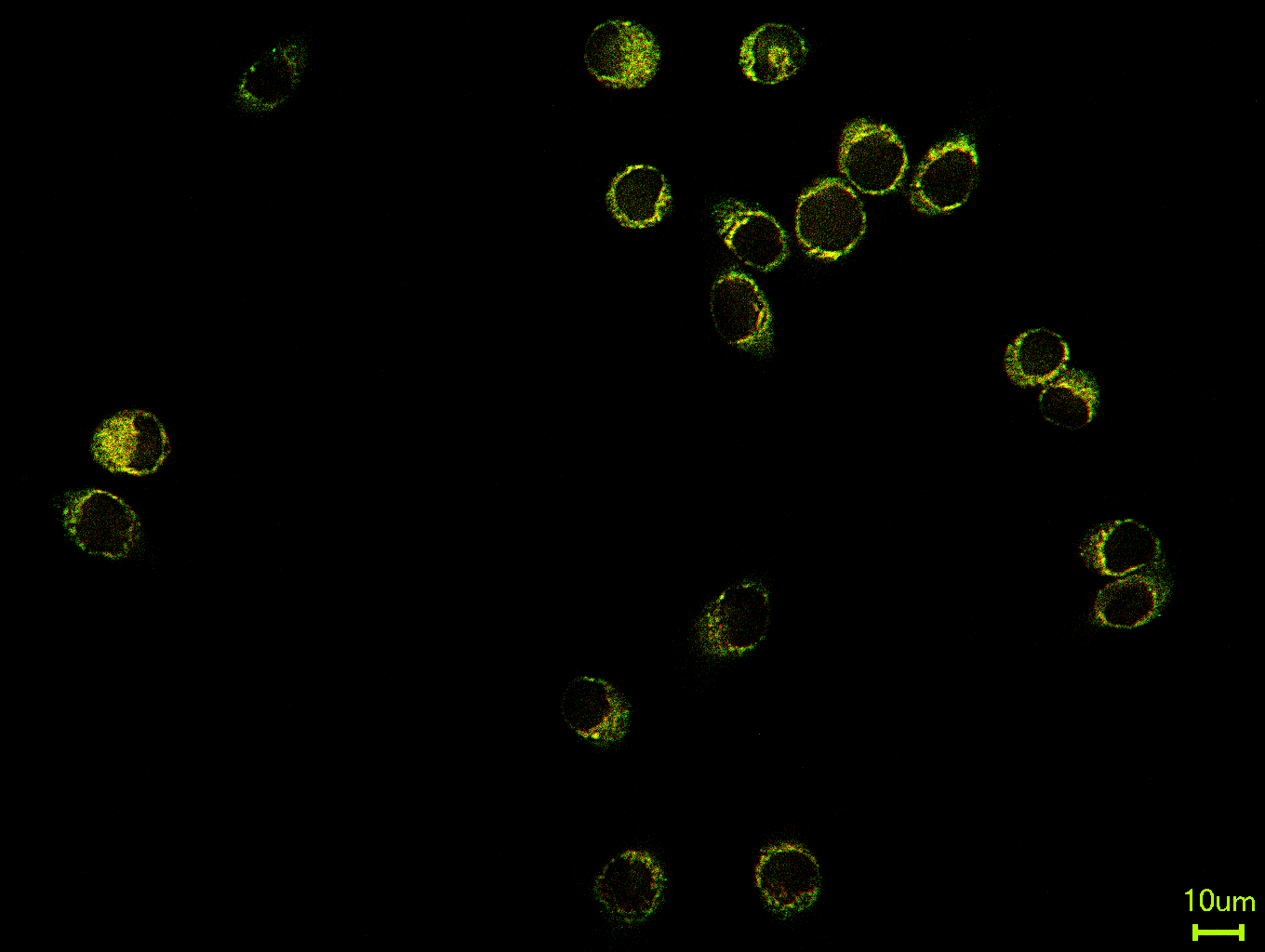

Supplement: Supplemental Information 5 — RAW264.7 were cultured in basic medium (RPMI 1640 containing 4.5 g/L glucose, 5% v/v FCS, 0.1% v/v ethanol) supplemented with docosahexaenoic acid (DHA) in a concentration of 15 µM for 72 h. Co-localization of TLR1 with GM1 was analyzed by indirect immunofluorescence microscopy. In the representative images TLR1 is labeled in green; GM1 is labeled in red. Scale bar represents 10 µm. [file peerj-06-4212-s006.png]

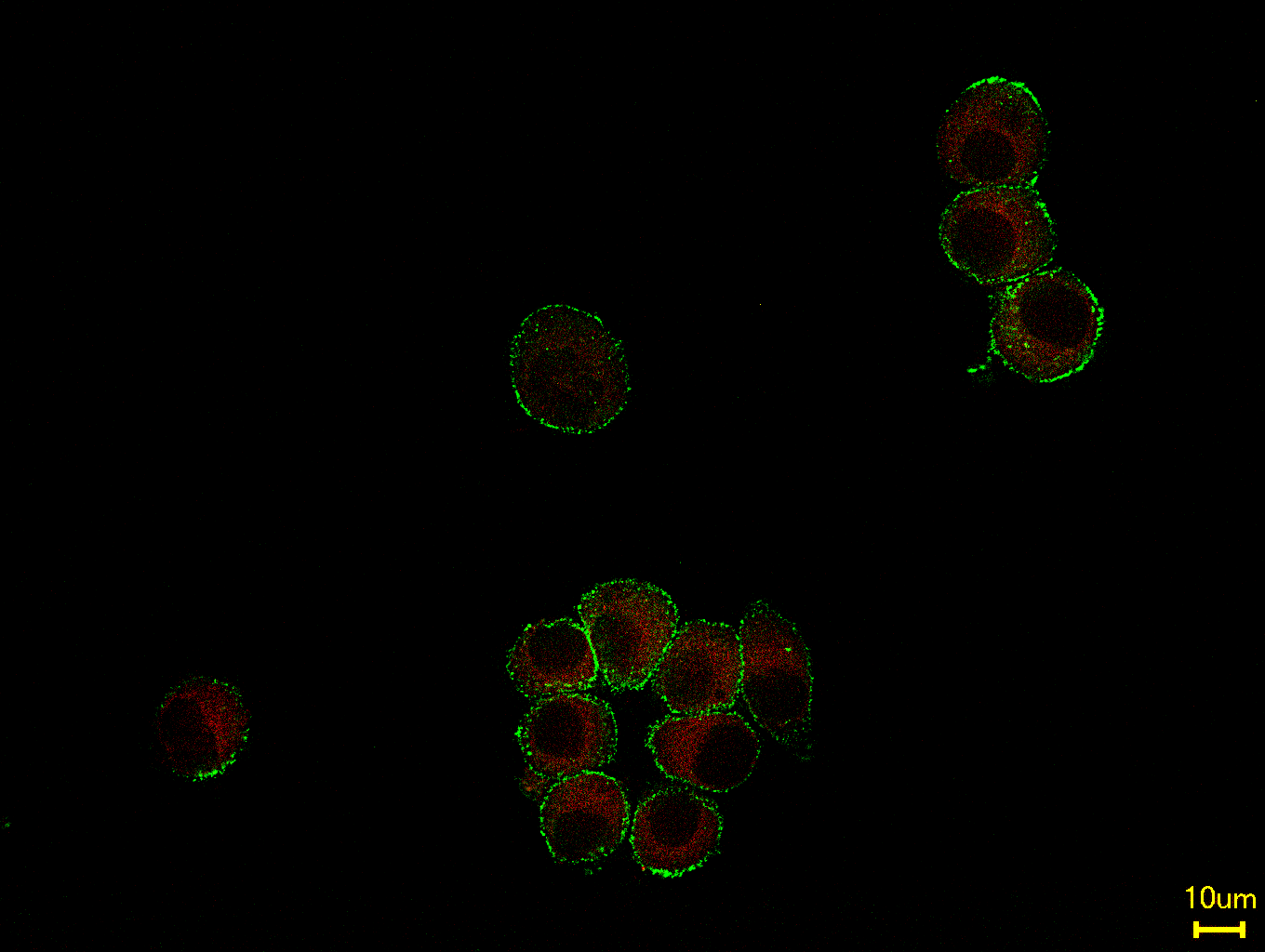

Supplement: Supplemental Information 6 — RAW264.7 were cultured in basic medium (RPMI 1640 containing 4.5 g/L glucose, 5% v/v FCS, 0.1% v/v ethanol) supplemented with arachidonic acid (AA) in a concentration of 15 µM for 72 h. Stimulation was performed by addition of LTA (0.5 µg/mL) to the culture medium in the last 24 h of incubation. Co-localization of TLR2 with GM1 was analyzed by indirect immunofluorescence microscopy. In the representative image TLR2 is labeled in green; GM1 is labeled in red. Scale bar represents 10 µm. [file peerj-06-4212-s007.png]

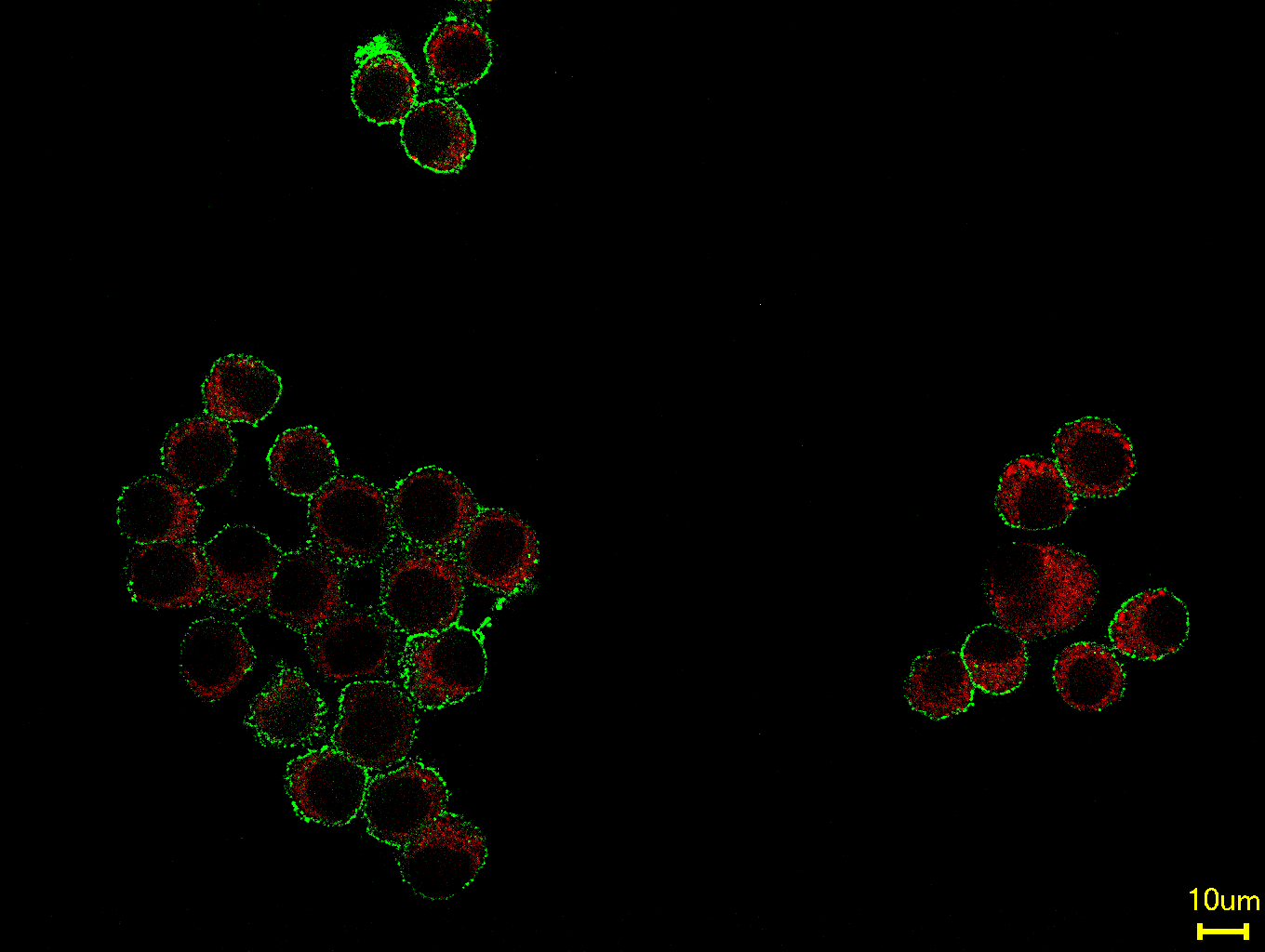

Supplement: Supplemental Information 7 — RAW264.7 were cultured in basic medium (RPMI 1640 containing 4.5 g/L glucose, 5% v/v FCS, 0.1% v/v ethanol) supplemented with arachidonic acid (AA) in a concentration of 15 µM for 72 h. Co-localization of TLR2 with GM1 was analyzed by indirect immunofluorescence microscopy. In the representative image TLR2 is labeled in green; GM1 is labeled in red. Scale bar represents 10 µm. [file peerj-06-4212-s008.png]

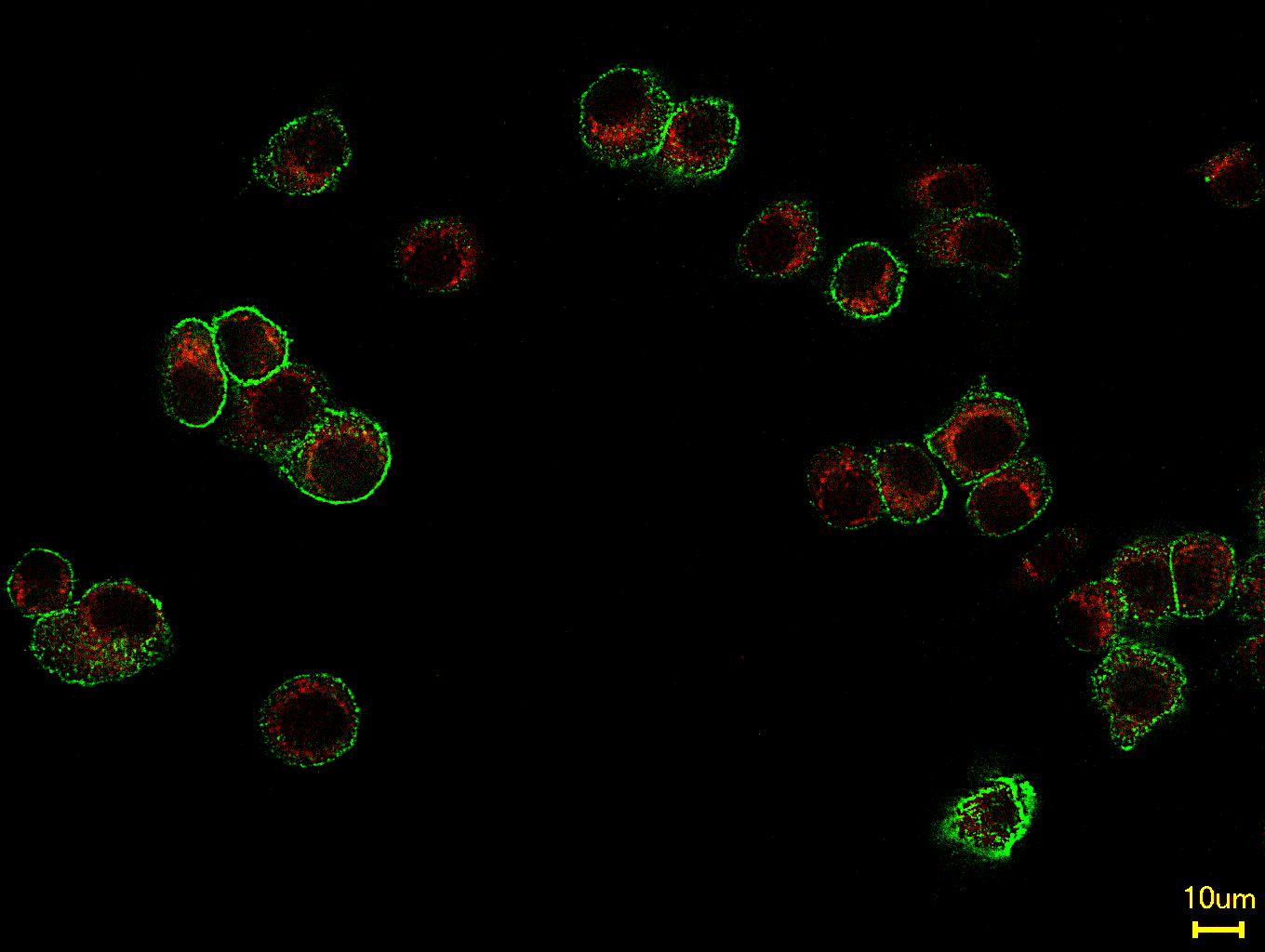

Supplement: Supplemental Information 8 — RAW264.7 were cultured in basic medium (RPMI 1640 containing 4.5 g/L glucose, 5% v/v FCS, 0.1% v/v ethanol) supplemented with docosahexaenoic acid (DHA) in a concentration of 15 µM for 72 h. Stimulation was performed by addition of LTA (0.5 µg/mL) to the culture medium in the last 24 h of incubation. Co-localization of TLR2 with GM1 was analyzed by indirect immunofluorescence microscopy. In the representative image TLR2 is labeled in green; GM1 is labeled in red. Scale bar represents 10 µm. [file peerj-06-4212-s009.png]

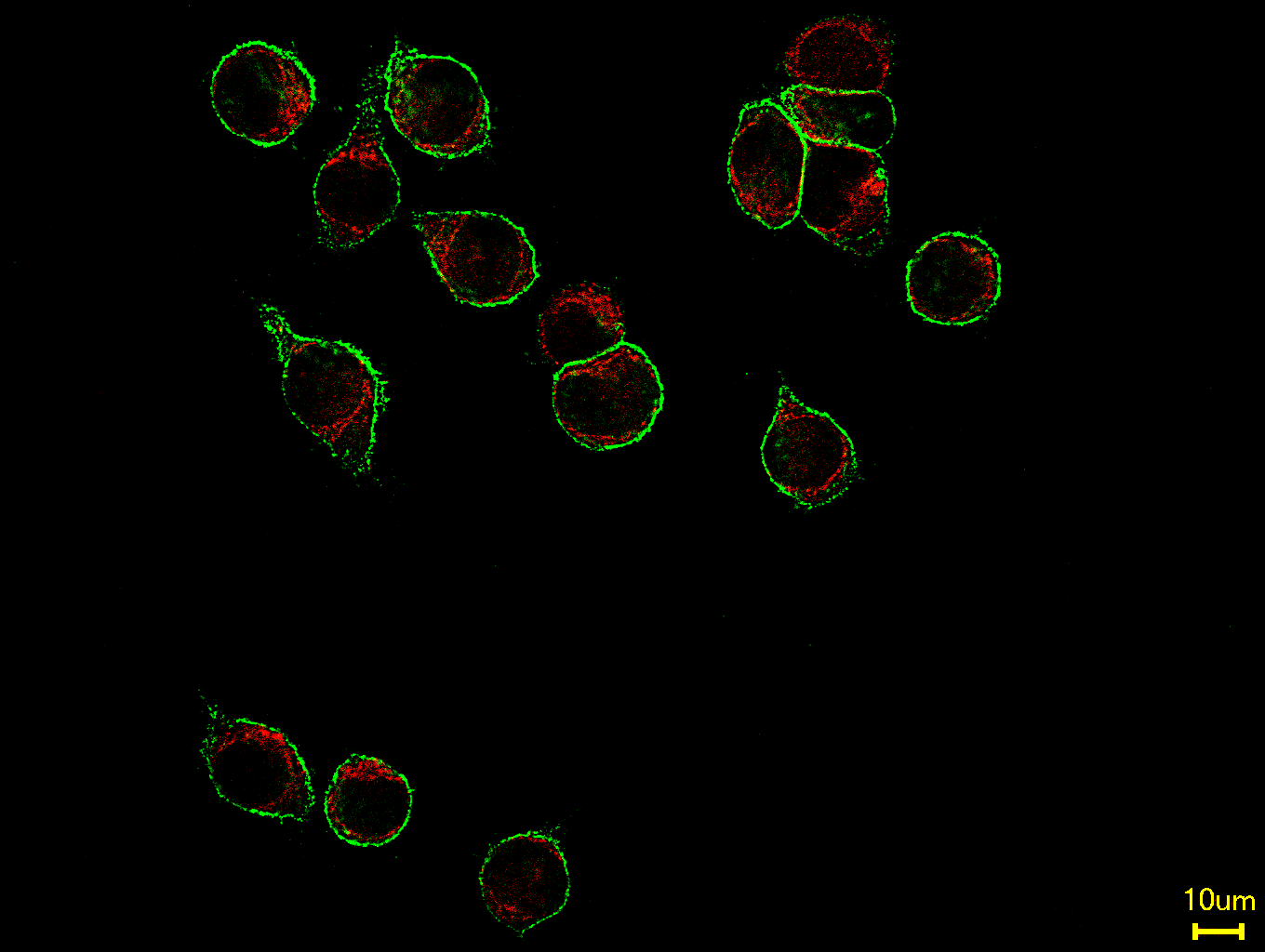

Supplement: Supplemental Information 9 — RAW264.7 were cultured in basic medium (RPMI 1640 containing 4.5 g/L glucose, 5% v/v FCS, 0.1% v/v ethanol) supplemented with docosahexaenoic acid (DHA) in a concentration of 15 µM for 72 h. Co-localization of TLR2 with GM1 was analyzed by indirect immunofluorescence microscopy. In the representative image TLR2, is labeled in green; GM1 is labeled in red. Scale bar represents 10 µm. [file peerj-06-4212-s010.png]

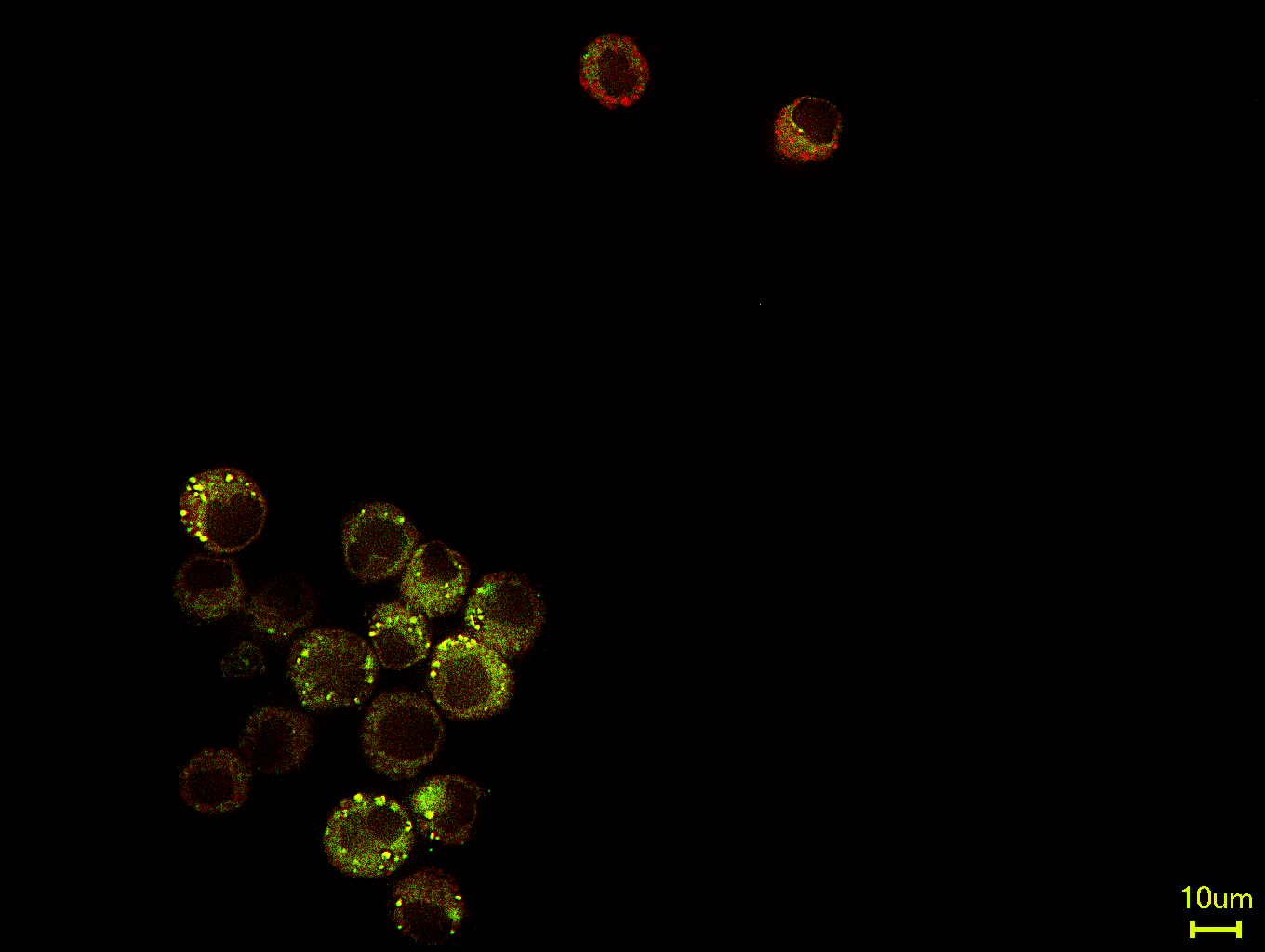

Supplement: Supplemental Information 10 — RAW264.7 were cultured in basic medium (RPMI 1640 containing 4.5 g/L glucose, 5% v/v FCS, 0.1% v/v ethanol) supplemented with arachidonic acid (AA) in a concentration of 15 µM for 72 h. Stimulation was performed by addition of LTA (0.5 µg/mL) to the culture medium in the last 24 h of incubation. Co-localization of TLR6 with GM1 was analyzed by indirect immunofluorescence microscopy. In the representative imags TLR6 is labeled in green; GM1 is labeled in red. Scale bar represents 10 µm. [file peerj-06-4212-s011.png]

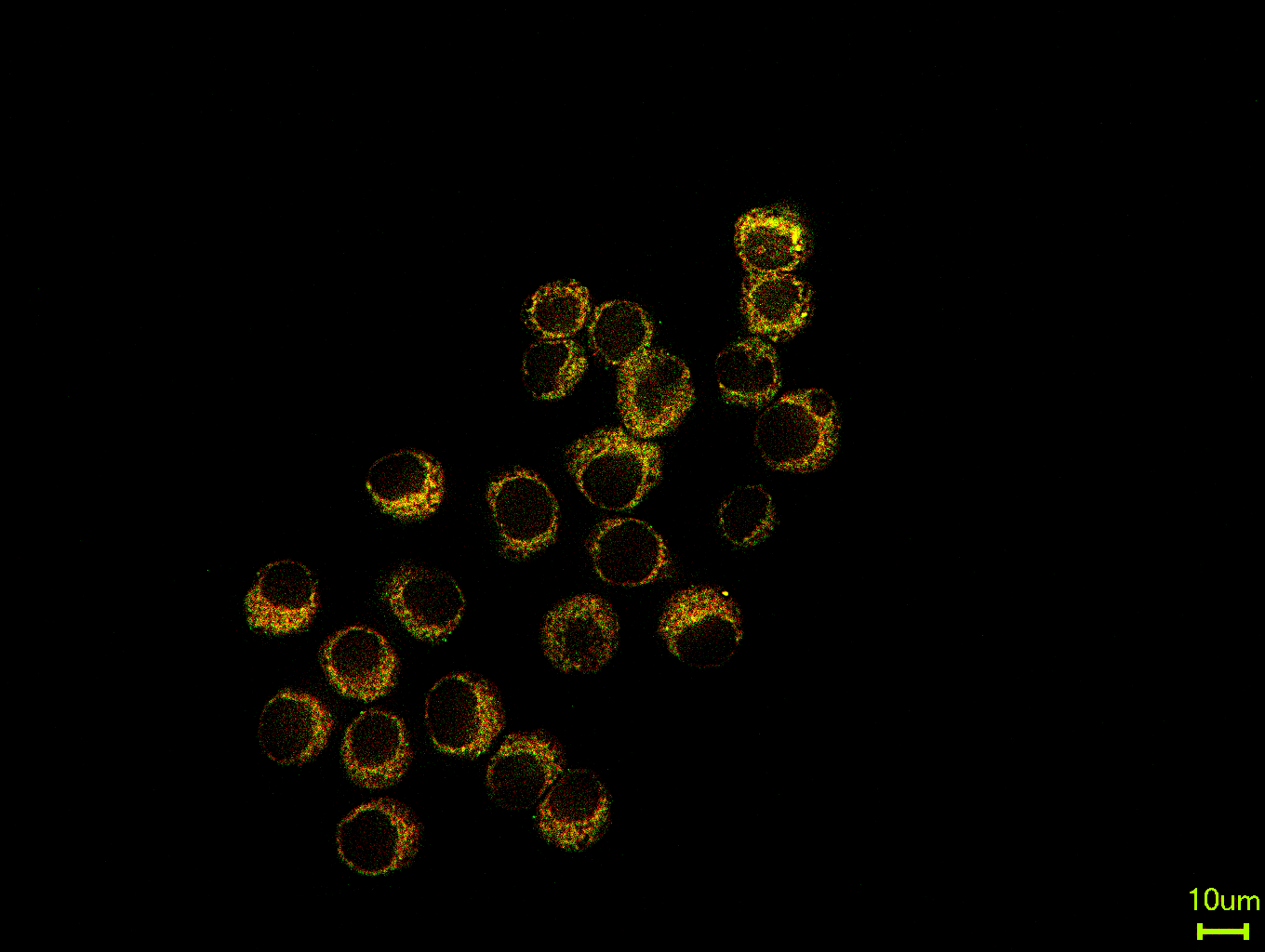

Supplement: Supplemental Information 11 — RAW264.7 were cultured in basic medium (RPMI 1640 containing 4.5 g/L glucose, 5% v/v FCS, 0.1% v/v ethanol) supplemented with arachidonic acid (AA) in a concentration of 15 µM for 72 h. Co-localization of TLR6 with GM1 was analyzed by indirect immunofluorescence microscopy. In the representative images TLR6 is labeled in green; GM1 is labeled in red. Scale bar represents 10 µm. [file peerj-06-4212-s012.png]

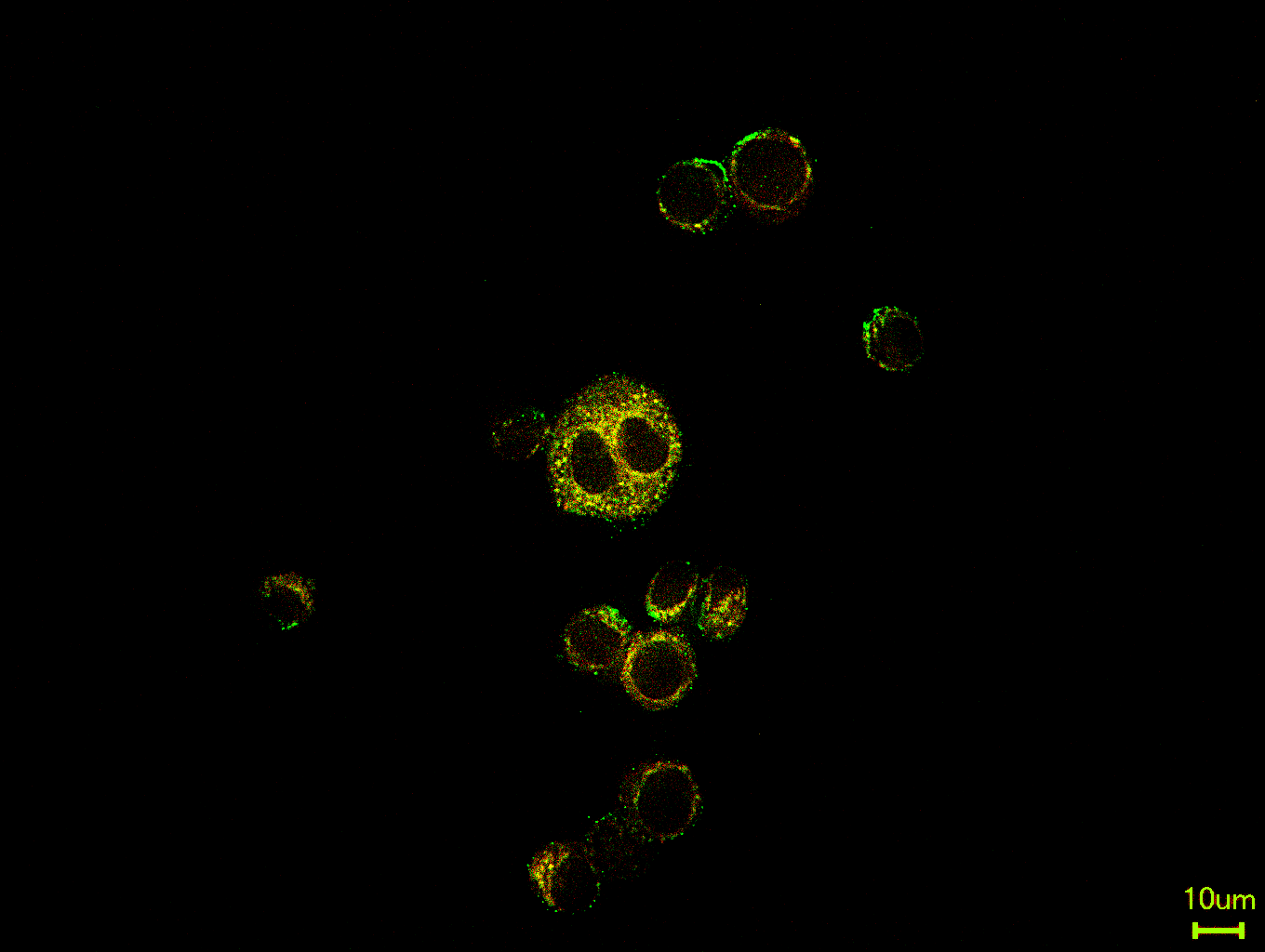

Supplement: Supplemental Information 12 — RAW264.7 were cultured in basic medium (RPMI 1640 containing 4.5 g/L glucose, 5% v/v FCS, 0.1% v/v ethanol) supplemented with docosahexaenoic acid (DHA) in a concentration of 15 µM for 72 h. Stimulation was performed by addition of LTA (0.5 µg/mL) to the culture medium in the last 24 h of incubation. Co-localization of TLR6 with GM1 was analyzed by indirect immunofluorescence microscopy. In the representative image TLR6 is labeled in green; GM1 is labeled in red. Scale bar represents 10 µm. [file peerj-06-4212-s013.png]

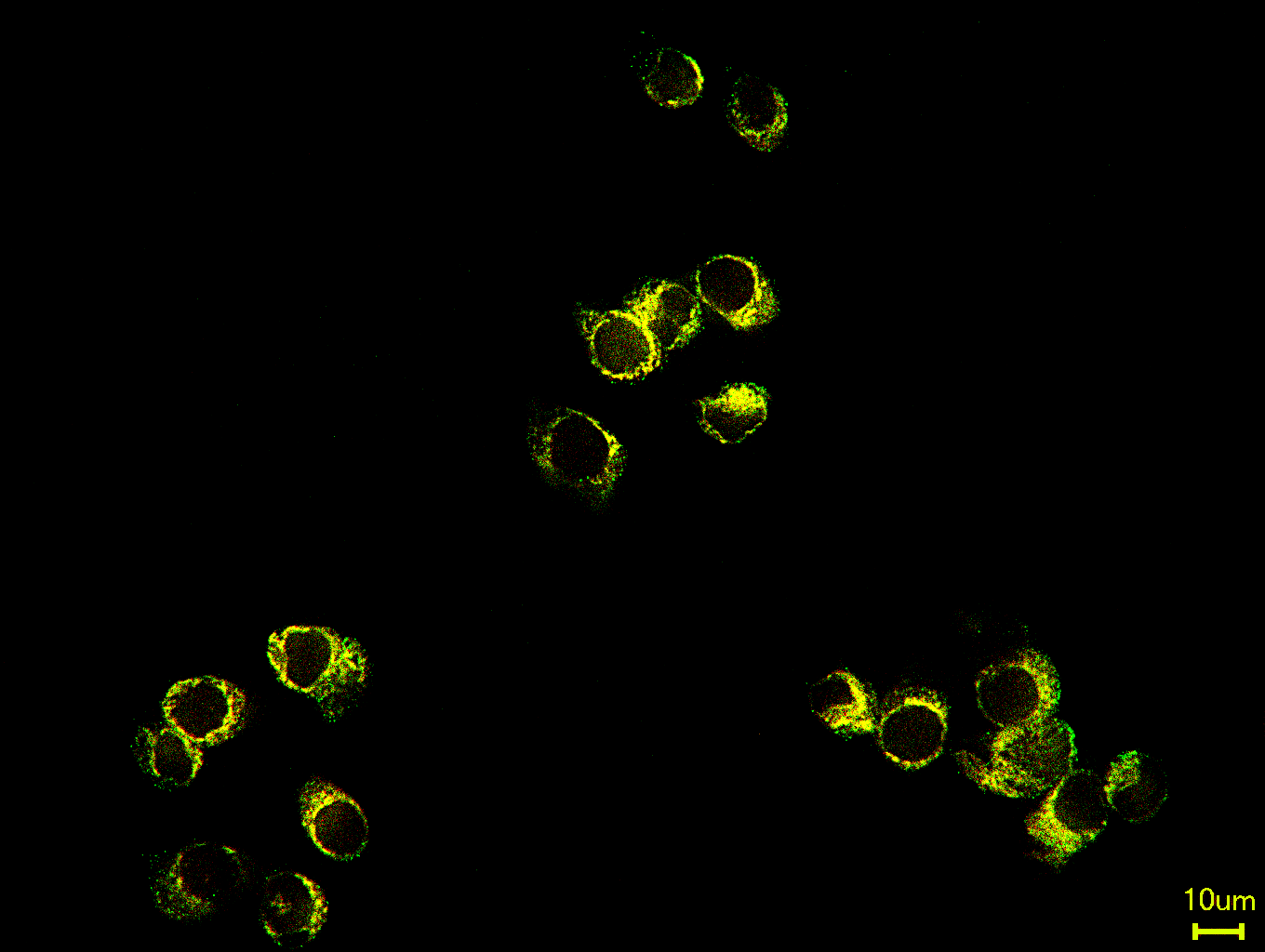

Supplement: Supplemental Information 13 — RAW264.7 were cultured in basic medium (RPMI 1640 containing 4.5 g/L glucose, 5% v/v FCS, 0.1% v/v ethanol) supplemented with docosahexaenoic acid (DHA) in a concentration of 15 µM for 72 h. Co-localization of TLR6 with GM1 was analyzed by indirect immunofluorescence microscopy. In the representative image TLR6 is labeled in green; GM1 is labeled in red. Scale bar represents 10 µm. [file peerj-06-4212-s014.png]

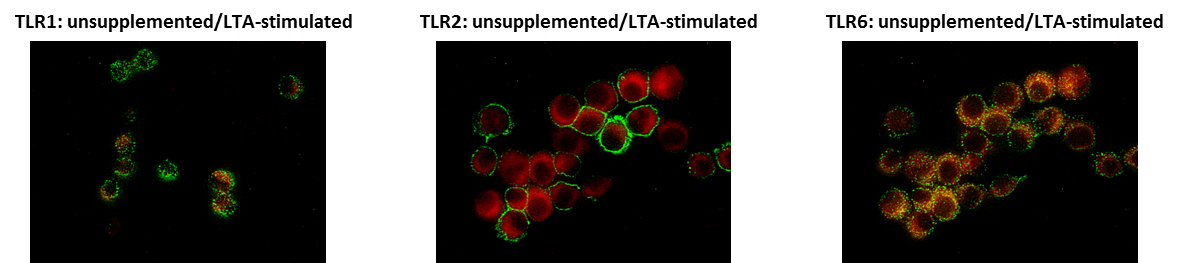

Supplement: Supplemental Information 14 — RAW264.7 were cultured in basic medium (RPMI 1640 containing 4.5 g/L glucose, 5% v/v FCS, 0.1% v/v ethanol). Stimulation was performed by addition of LTA (0.5 µg/mL) to the culture medium for 15 min. Co-localization of TLR1, TLR2 or TLR6 with GM1 was analyzed by indirect immunofluorescence microscopy. In the representative images the TLRs are labeled in green; GM1 is labeled in red. [file peerj-06-4212-s015.png]
